# Supplementary material for: Surface Defects Improved SERS Activity of Nanoporous Gold Prepared by Electrochemical Dealloying
Source: Nanomaterials (Basel). 2022 Dec 31;13(1):187. doi: 10.3390/nano13010187 (PMC9824599; doi:10.3390/nano13010187)
Supplement: Supplementary file 1 [file nanomaterials-13-00187-s001.zip › nanomaterials-2104236-supplementary.pdf]

Article

Surface Defects Improved SERS Activity of Nanoporous Gold Prepared by Electrochemical Dealloying

Ling Zhang [1,*], Zhiyu Jing [1], Zhexiao Li [1] and Takeshi Fujita [2]

1 School of Optical-Electrical and Computer Engineering, University of Shanghai for Science and Technology, Shanghai 200093, China; 201310032@st.usst.edu.cn (Z.J.); 202310295@st.usst.edu.cn (Z.L.)
2 School of Environmental Science and Engineering, Kochi University of Technology, Kochi 782-8502, Japan; fujita.takeshi@kochi-tech.ac.jp
* Correspondence: lzhang@usst.edu.cn

1. Surface area to volume ratio (SVR) estimation.

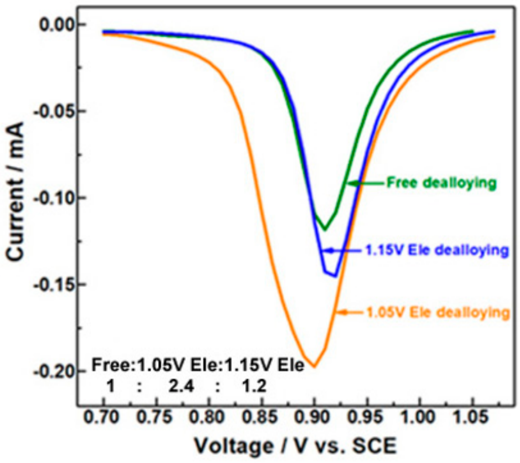

Figure S1. Reduction curves of Au oxide from NPG-Free, NPG-1.05V Ele and NPG-1.15V Ele.

A three-electrode electrochemical cell was used for the electrochemical experiments. Ag/AgCl(3.0M KCl) was used as reference electrode and Pt sheet was used as counter electrode. A glassy carbon(GC) electrode (4 mm in diameter) was used as the substrate electrode for located NPG films.The GC electrode was first polished with alumina powder, and then sonication in deionized water. after dried in the air, 100 nm thick NPG films was fixed on the surface with 0.2 μL Nafion solution (5%). The surface area was estimated from cyclic voltammetry (CV) curve that obtained in 0.5M H_2SO_4 aqueous solution (electrolyte).

Figure S1 shows the reduction curves of Au oxide, and we considered single layer of Au atoms was oxided and the reducted. We can calculated the total amount of the electron with the formula:

$$Q = UI,$$

where Q is the transferred electron, I is the current at corresponding potential U.

Since, the electrical quantity(q) that required for single-atomic layer oxidation of gold is around 370-390μc/cm^2, the surface area of NPG thin film (A)can be estimated with A=Q/q.

The calculated surface area of NPG-free, NPG-1.05 V Ele and NPG-1.15 V Ele are 0.4651cm^2 , 0.5736cm^2 and 1.1cm^2, respectively. According to the equation, the surface area of NPG is proportional to the transferred electron which is calculated with the CV curve. Also we can get the ratio of surface area between different NPG films directly from the area enclosed with the reduction curve.

Additionally, the surface-to-volume ratio (SVR) was calculated by SVR = A/V, where V is the volume of NPG film on the GC electrode. Since the density of the three samples

are identical, the volume of them are also the same. Thus, we can get the SVR of NPG-free: NPG-1.05 V Ele : NPG-1.15 V Ele is 1:2.4:1.2,which is as the same as the ratio of surface area.

2. Surface O analysis

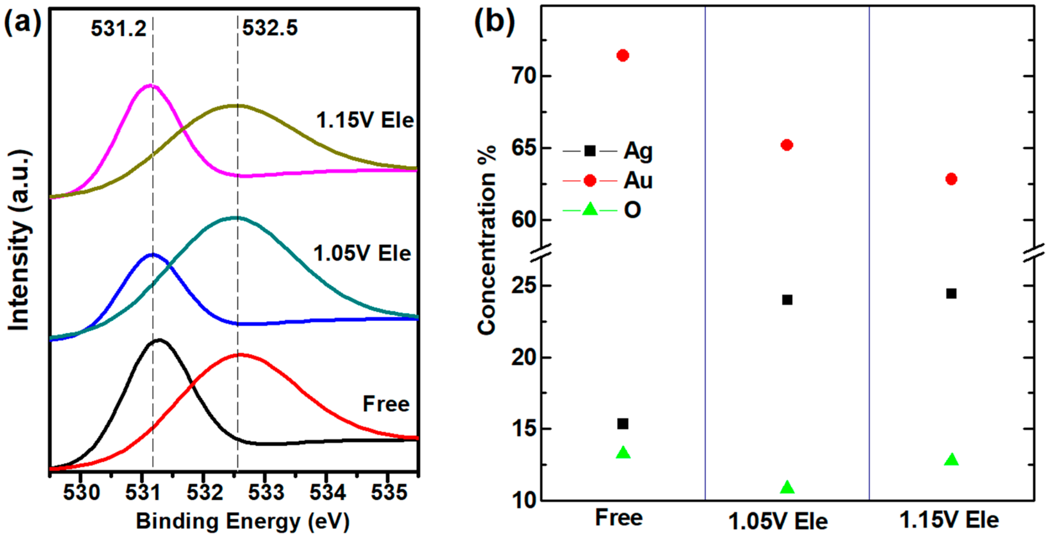

Figure S2. (**a**) Gaussian fitting of O 1s spectra for the three selected samples, and (**b**) Elements concentration near the surface of the three selected samples.

As shown in Figure S2a, the peak at 531.2 is associated to oxygen ions in crystal lattice (O-latt), and the peak at 532.5 eV is attributed to surface adsorbed oxygen (O_2-ads). Compare the relative intensity of O-latt with O_2-ads, the O-latt of NPG-1.05V Ele is less than that of NPG-Free and NPG-1.15 V Ele. Oppositely, O_2-ads of NPG-1.05V Ele is more than that of NPG-Free and NPG-1.15 V Ele. The surface content difference of O pieces indicate that an applicable voltage can inhibit surface oxidation as well as increase surface area, all of which lead to better SERS activity. Moreover, the applied voltage can remain more silver elements near the surface during the formation of nanopore structure. As shown in Figure S2b, the surface silver content of NPG-1.05V Ele and NPG-1.15V Ele is around 25 at.%, while that of NPG-free is only 15 at.%, although the average silver concentration of the three samples are basically identical.

3. SEM images and SERS spectra of NPG with 3 at.% residual silver.

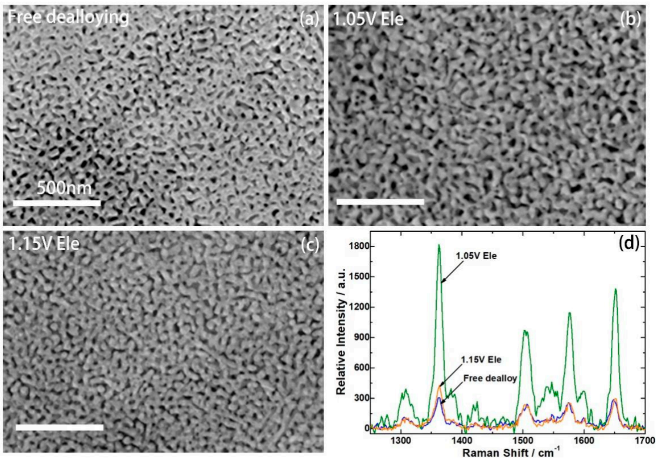

Figure S3. SEM images and SERS spectra of NPG with similar residual silver content (3 at.%) that prepared at different conditions. SEM images of the three samples (a) free dealloyed NPG with the

ligament and pore size around 21±4 nm, (b) electrochemical dealloyed NPG at 1.05 V with the ligament and pore size of 40±8 nm, and (c) electrochemical dealloyed NPG at 1.15 V with the ligament and pore size of 28±5 nm, (d)SERS spectra of the three samples with 3 at.% residual silver shown in (a), (b) and (c).

Figure S3 shows the SEM images and SERS spectra of NPG with 3 at.% residual silver that prepared at different conditions. With similar silver concentration, NPG prepared with 1.05 V Ele-dealloying appears much better SERS enhancement than the NPG prepare with free daealloying and 1.15 V Ele-dealloying.

4. NPG prepared with $Ag_{80}Au_{20}$ alloy.

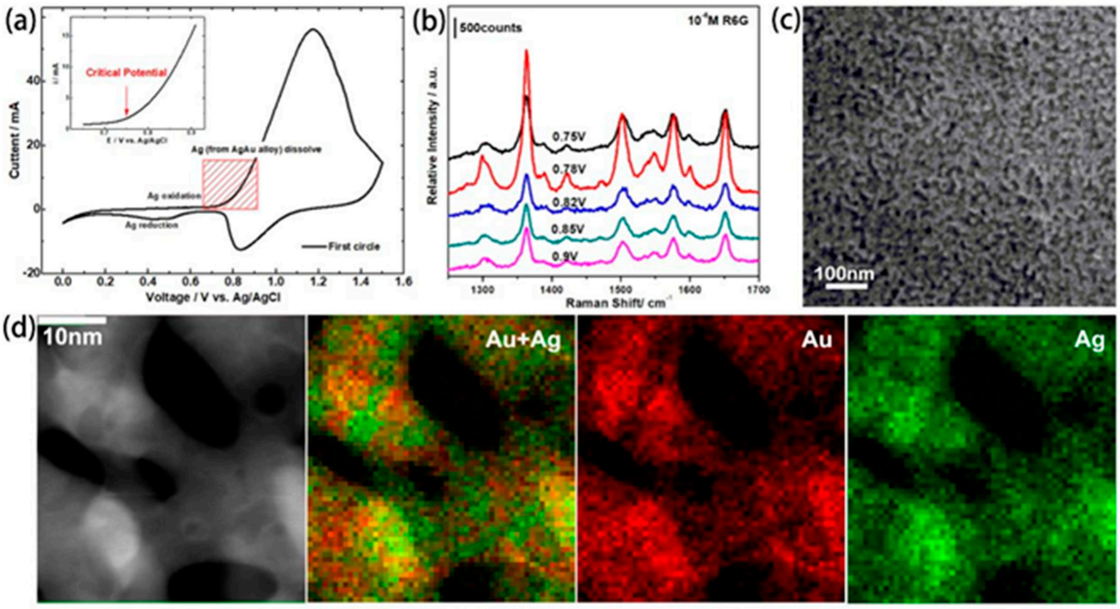

Figure S4. NPG prepared with $Ag_{80}Au_{20}$ alloy and its properties.(a) Experimental cyclic voltammetry curve of $Ag_{80}Au_{20}$ in 2M HNO_3 acid solution (scan rate: $100mVs^{-1}$).(b) Strongest SERS spectra of NPG prepared at different constant potential. (c) SEM image of NPG prepared by electrochemical dealloying at 0.78V for 1000s, which exhibits the best-improved SERS enhancement. (d) Chemical analysis of NPG (0.78V Ele) that shows in (c).

Figure S4a is the experimental cyclic voltammetry curve of $Ag_{80}Au_{20}$ in 2M HNO_3 acid solution, and the critical potential is around 0.75 V. Figure S4b shows the strongest SERS spectra of NPG prepared at different constant potentials, and the one prepared with the constant potential slightly higher than the critical potential (0.78V) exhibited the best SERS enhancement. Figure S4c is the SEM image of the NPG prepared at 0.78V, and the chemical analysis of selected region is shown in Figure S4d.
